# Review of the subgenus *Polyphylla (Granida)* from continental Asia (Coleoptera, Scarabaeidae, Melolonthinae)

**DOI:** 10.3897/zookeys.102.1148

**Published:** 2011-06-02

**Authors:** Richard Sehnal, Aleš Bezděk

**Affiliations:** 1 V Kopečku 140, CZ–289 01 Velenice, Czech Republic; 2 Biology Centre ASCR, Institute of Entomology, Branišovská 31, CZ-370 05 České Budějovice, Czech Republic

**Keywords:** new species, new locality records, Scarabaeidae, Melolonthinae, Melolonthini, *Polyphylla*, *Granida*, mainland Asia

## Abstract

A review of *Polyphylla* Harris, 1841, species belonging to the subgenus *Granida* Motschulsky, 1861, from continental Asia is presented. One new species is described from Thailand: *Polyphylla (Granida) simoni* **sp. n.** *Polyphylla (Granida) nikodymi* de Wailly, 1993, is recorded from Thailand for the first time. *Polyphylla (Granida) minor* Nomura, 1977, is recorded from Yunnan (China) for the first time. The previously unknown female of *Polyphylla (Granida) phongsali* Zídek, 2006, is described.

## Introduction

The subgenera *Granida* Motschulsky, 1861, and *Grananoxia* Brenske, 1890, of the genus *Polyphylla* Harris, 1841, form a pair of subgenera distinguished from other Eurasian subgenera by unequal tarsal claws in males. The basal tooth of the inner claw on the protarsus is distinctly longer than that of the outer claw, whereas the meso- and metatarsal claws bear more robust basal teeth on the outer claws. As far as it is known, tarsal claws in females are equal. Members of the subgenus *Grananoxia* differ from *Granida* species by their nearly unicolor pale brown body and entire surface of vertex and pronotum covered with pale, long erect setae (Li and Yang 1997). Species of the subgenus *Granida* are characterized by the surface of vertex and pronotum having a rather complex scaly pattern (e.g., Zídek 2006).

In the literature, the pattern of elytral sculpture (four scaly longitudinal stripes on each elytron) has also been used as a suitable delimiting character of the subgenus *Granida* (e.g., de Wailly 1993). However, this character was rejected by Zídek (2006) and recently also by Kobayashi and Chou (2008) and Keith (2010). Zídek (2006) described *Polyphylla (Granida) phongsali*, currently the only known *Polyphylla (Granida)* species with maculate elytral pattern, while the elytra of *Polyphylla (Granida) parva* Kobayashi & Chou, 2008, bear scaly stripes being strongly reduced, with only sutural and lateral stripes visible. It should be noted, that some other characters generally used for the delimitation of Eurasian subgenera of the genus *Polyphylla* (e.g., number of teeth on the outer margin of protibia), were found to be extremely variable in Nearctic members of *Polyphylla*, even within particular species (Young 1988).

Eight species of the subgenus *Granida* are currently recognized. De Wailly (1993) and Bezděk (2006) listed six species, and two additional were described by Zídek (2006) and by Kobayashi and Chou (2008). Five of them are rather well known species distributed in Japan: *Polyphylla (Granida) albolineata* (Motschulsky, 1861) and *Polyphylla (Granida) schoenfeldti* Brenske, 1890, and Taiwan: *Polyphylla (Granida) taiwana* (Sawada, 1950), *Polyphylla (Granida) minor* Nomura, 1977, and *Polyphylla (Granida) parva* Kobayashi & Chou, 2008. The remaining three continental Asian species are rare and known from a very limited number of specimens. The *Polyphylla (Granida) jessopi* de Wailly, 1993, and *Polyphylla (Granida) phongsali* Zídek, 2006, were described from single male specimens from China and Laos, respectively. *Polyphylla (Granida) nikodymi* de Wailly, 1993, was known only from five type male specimens from Myanmar.

Recently, the authors had the opportunity to study several specimens of the subgenus *Granida* collected by Czech entomologists in China, Laos and Thailand. Examination of this material allowed us to describe one new species, to describe previously unknown female of *Polyphylla (Granida) phongsali*, and to define the geographic distribution of *Polyphylla (Granida) nikodymi* and *Polyphylla (Granida) minor*.

Since the previously known continental Asian *Polyphylla (Granida)* species were described recently and their descriptions are rather detailed, the authors have decided to mention only the important diagnostic characters of these species.

## Material and methods

The following abbreviations (after Arnett et al. 1993) identify the collections housing the material examined (curators names are in parentheses):

ABCC	Czech Republic, České Budějovice, Aleš Bezděk collection;

BMNH	United Kingdom, London, Natural History Museum (Malcolm Kerley, Maxwell Barclay);

DKCC	France, Chartres, Denis Keith collection;

JZCP	Czech Republic, Praha, Jiří Zídek collection;

MNCP	Czech Republic, Praha, Milan Nikodým collection;

NMPC	Czech Republic, Praha, National Museum (Natural History) (Jiří Hájek);

PFHC	Czech Republic, Hradec Králové, Pavel Filip collection;

PPCB Czech Republic, Brno, Petr Pacholátko collection;

RSCV	Czech Republic, Velenice, Richard Sehnal collection;

Specimens of the newly described species are provided with one red printed label: “*Polyphylla simoni*, HOLOTYPUS [PARATYPUS], [type specimen number], ♂, R. Sehnal & A. Bezděk det. 2009”.

Exact label data are cited for type specimens. Authors’ remarks are in brackets: [p] – printed; [h] – handwritten. Labels are separated by double slash “//”.

## Taxonomy

### Polyphylla (Granida) simoni

Sehnal & Bezděk sp. n.

urn:lsid:zoobank.org:act:5BB817CC-4399-4DED-BDE3-CFAF2DE8B6BC

http://species-id.net/wiki/Polyphylla_(Granida)_simoni

Figs 1–4 

#### Type locality.

“N Thailand, 100 km NE of Nan, Doi Phu Kha N.P.”.

#### Type material examined.

Holotype (male), labeled: “N Thailand, 100 km NE of Nan, Doi Phu Kha N.P., 20.-25.IV.2004, Filip Pavel lgt. [p]”, in BMNH; paratypes Nos. 1–5 (all males), same data, PT Nos. 1–3 in RSCV, PT No. 4 in NMPC, PT No. 5 in PFHC.

#### Description of holotype.

**Figures 1–4.**
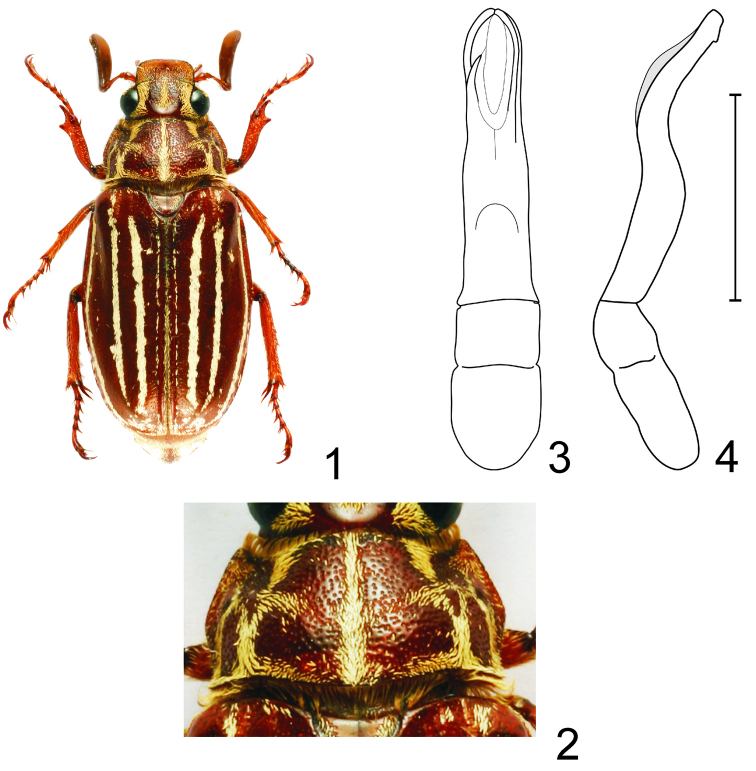
*Polyphylla (Granida) simoni* sp. n. **1** Habitus of holotype male (length 22.0 mm), dorsal view **2** Detail of pronotum, dorsal view **3** Male genitalia, dorsal view **4** The same, right lateral view, shaded area indicates overlapping part of left paramere. Scale bar: 5 mm for Figs 3–4.

Male, body length 22.0 mm excluding pygidium. Body elongate, moderately convex. Surface color chestnut brown, pronotum very slightly darker (Fig. 1). Dorsal surface of head, pronotum and scutellum covered with whitish to pale ochrous scales, elytra with whitish scales. Head appendages, legs (except of femora) and ventral surface of abdomen covered with short, whitish to pale ochrous setae. Pro-, meso- and metasternum as well as femora with long pale ochrous hair-like setae.

Labrum deeply bilobed with several erect setae laterally. Clypeus transverse with anterior margin considerably upturned, anterior angles broadly rounded, sides very slightly convergent posteriad; surface with coarsely, dense, laterally somewhat confluent punctures; scales denser and erect along anterior and lateral margins, posteriorly less dense and recumbent. Frontoclypeal suture present, forming an uninterrupted narrow ridge. Frons coarsely, irregularly punctured. Pale ochrous scales on frons form three stripes, medial longitudinal stripe separated from lateral stripes by coarsely punctured areas. Vertex impunctate and shiny. Canthus narrow, reaches to about half of eye width, with pale ochrous erect setae. Angle between lateral side of clypeus and canthus obtuse (in view from above). Antenna with ten antennomeres, club heptamerous, gently curved outwards, two times longer than shaft. Scapus dilated apically and covered with narrow brush of moderately long erect setae, pedicellus short and stout, about as long as wide, antennomere 3 slender, with three erect setae, as long as basal antennomeres combined. Terminal maxillary palpomere sparsely covered with short erect setae.

Pronotum transverse, convex, widest approximately at middle. Lateral margins bisinuate, anterior angles prominent with rounded apex, posterior angles obtusely angulate with somewhat upturned apex. Anterior margin thinly bordered. Basal border interrupted medially. Surface of pronotum rugged, with complex scaly pattern (Fig. 2).

Scutellum parabolic, with disc slightly impressed and impunctate, lateral sides covered with scales, apex broadly rounded.

Elytra nearly parallel-sided in basal half, rounded apically, moderately convex. Surface coarsely irregularly punctuate, covered with whitish scales forming four longitudinal stripes on each elytron plus one short longitudinal row of few isolated patches arising on humeral umbone. Longitudinal stripes with poorly defined edges. Beetle macropterous, capable of flying.

Ventral surface of thorax densely covered with long, erect setae. Abdominal sternites with dense, short, recumbent setae, anterior margin impunctate. Pygidium triangulate, broadly rounded apically, densely covered with recumbent scales, nearly impunctate and with only few isolated setae along midline.

Pro- and mesofemora densely, irregularly punctuate, with long erect setae. Setae of metafemora somewhat sparser and shorter. Protibia bidentate, covered with sparse, short, setae, terminal spur inserted against basal tooth. Meso- and metatibia very slightly expanded apically, with transversal carina medially armed with 3–4 short thick bristles. Surface of meso- and metatibia covered with sparse, short, recumbent setae, mixed with long and erect setae on inner sides. Tarsal claws with distinct basal tooth ventrally, unequal in all legs. Protarsus with distinctly longer basal tooth of inner claw, whereas meso- and metatarsi with more robust basal teeth on outer claws.

Male genitalia. Parameres fused basally for more than half of length, nearly two times longer than phallobase (Fig. 3, arcuate in lateral view, with a small ventral tooth apically (Fig. 4).

Female unknown.

#### Variability.

The paratypes slightly vary in body length (20.0–23.0 mm, excluding pygidium), otherwise they are very similar to the holotype.

#### Etymology.

The species is named in honor of Šimon, son of the first author.

#### Collecting method.

All specimens were collected at light.

#### Distribution.

NE Thailand (Fig. 20).

#### Diagnosis.

This species belongs to a group of *Granida* species with well-defined scaly stripes on the elytra. *Polyphylla (Granida) albolineata*, *Polyphylla (Granida) schoenfeldti* and *Polyphylla (Granida) taiwana* are rather large (27–32 mm) and with unidentate protibia in males. *Polyphylla (Granida) simoni* sp. n. is thus similar mainly to *Polyphylla (Granida) nikodymi* from mainland Asia and *Polyphylla (Granida) minor* from Taiwan and China. These species are easily separated by the shape of the male genitalia. Parameres bear a small tooth subapically in *Polyphylla (Granida) simoni* sp. n. (see in the lateral view), while this small tooth is located much more basally in *Polyphylla (Granida) nikodymi* and *Polyphylla (Granida) minor* (compare Fig. 4 and Fig. 7). Moreover, the antennomere 3 is long and slender and more than three times longer than antennomere 2 in *Polyphylla (Granida) simoni*, while it is rather stout and twice as long as antennomere 2 in *Polyphylla (Granida) nikodymi* and *Polyphylla (Granida) minor*.

### Polyphylla (Granida) minor

Nomura, 1977

http://species-id.net/wiki/Polyphylla_(Granida)_minor

Figs 5–7

Polyphylla (Granida) minor  Nomura, 1977: 104.

#### Type locality.

“Wushe, Hotso, Taiwan”.

#### Type material not examined.

#### Additional material examined.

Formosa (Tchaj-wan), Nantou, Wushe, 1.6.-6.6.2002, Jar. Dalihod leg., 1 male in RSCV; Formosa (Tchaj-wan), Nantou, Wushe, 4.6.-6.6.2004, Jar. Dalihod leg., Jana Dalihodová Baštová leg., 2 males in RSCV; China, Yunnan prov., Kunming – Xishan, 19. 5. 1993, L. Bocák lgt., 1 male in PPCB.

#### Diagnosis.

*Polyphylla (Granida) minor* and *Polyphylla (Granida) nikodymi* share similar shape of antennomere 3 (rather short, only twice as long as antennomere 2 and with distinct anterodistal tooth). These species are easily separated by the shape of the male genitalia Figs 6–7 and Figs 9–10) and by the scaly pattern on pygidium (the pygidium is impunctate and bare along midline in *Polyphylla (Granida) minor*, while it is entirely covered with recumbent scales in *Polyphylla (Granida) nikodymi*).

**Figures 5–7.**
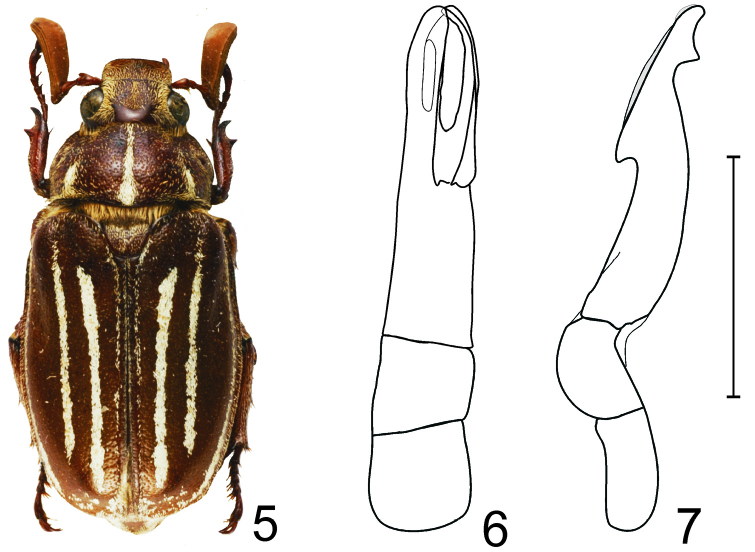
*Polyphylla (Granida) minor* **5** Habitus of male (Yunnan, China, length 19.5 mm), dorsal view **6** Male genitalia, dorsal view **7** The same, right lateral view, shaded area indicates overlapping part of left paramere. Scale bar: 5 mm for Figs 6–7.

#### Distribution.

Taiwan. Recorded from Yunnan province of China for the first time.

#### Remarks.

The specimen from Yunnan slightly differs from those from Taiwan by the shape of whitish scales on pronotum and elytra that are slightly broader. No relevant differences were found in the shape of male genitalia. If these morphologic characters were constant in other specimens coming from the same area, it would be reasonable to assume a subspecific status of the population from Yunnan. However, the material available is insufficient to decide whether such differences fall within the intersubspecific variability.

Although we were not able to study type material of *Polyphylla (Granida) minor*, all three males from Taiwan examined by us were collected from the type locality of this species.

### Polyphylla (Granida) nikodymi

de Wailly, 1993

http://species-id.net/wiki/Polyphylla_(Granida)_nikodymi

Figs 8–10

Polyphylla (Granida) nikodymi  de Wailly, 1993: 13.

#### Type locality.

“Birmanie, Süd-Ost”.

#### Type material examined.

Paratype (male), labeled: “Birmanie, Süd-Ost, 10.V.1990 [h] // PARATYPUS [p, red label] // Polyphylla (Granida) nikodymi De Wailly 1994 [h, red label]”, in NMPC; paratype (male), labeled: “Birmanie, Süd-Ost, 10.V.1990 [h] // PARATYPUS [p, red label]”, in PPCB.

#### Additional material examined.

THAILAND NE, Loei prov., Phu Rua N.P. 1100m, 17˚30′N, 101˚21′E, 6.-9.iv.1999, D. Hauck leg., 1 male in PPCB.

#### Diagnosis.

For separation from related species, see diagnosis of *Polyphylla (Granida) minor*. Male genitalia as in Figs 9–10.

**Figures 8–10.**
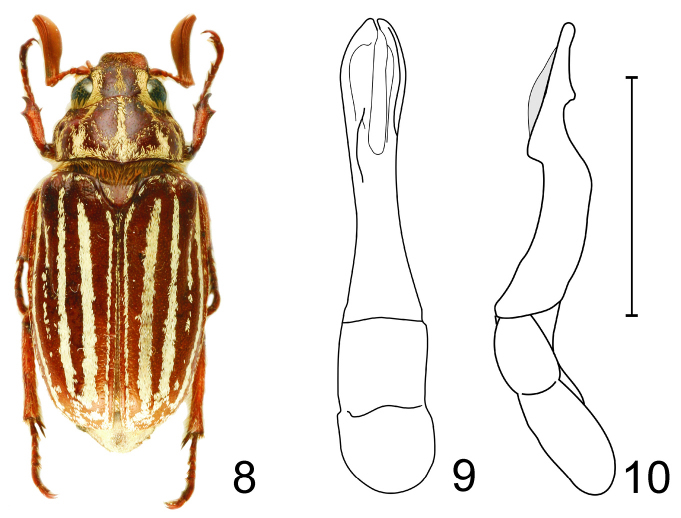
*Polyphylla (Granida) nikodymi* **8** Habitus of paratype male (length 22.5 mm), dorsal view **9** Male genitalia, dorsal view **10** The same, right lateral view, shaded area indicates overlapping part of left paramere. Scale bar: 5 mm for Figs 9–10.

#### Distribution.

Southeast Myanmar, first record for Thailand.

#### Remarks.

De Wailly (1993) wrote that antennomere 3 is long and slender. However, judging from the material available to us, the antennomere 3 is relatively short and rather stout (in comparison to other *Granida* members) with an anterodistal tooth, and only twice as long as antennomere 2.

Each specimen from the type series bears only a vague handwritten locality label “Birmanie, Süd-Ost”. Thus, the specimen from NE Thailand is the first specimen with exact locality data and it is the first record of this species for Thailand.

Paratypes of *Polyphylla (Granida) nikodymi* are deposited in PPCB and NMPC (see also Bezděk and Hájek 2010); none of them is housed in the collection of David Král (Prague, Czech Republic) as was erroneously stated by de Wailly (1993).

### Polyphylla (Granida) jessopi

de Wailly, 1993

http://species-id.net/wiki/Polyphylla_(Granida)_jessopi

Figs 11–15

Polyphylla (Granida) jessopi  de Wailly, 1993: 12.

#### Type locality.

“China, Foochow”.

#### Type material examined.

Holotype (male), labeled: “CHINA, Foochow [p], vi. 1936 [h], M. S. Yang [p, white label] // next to Polyphylla nov. sp. [h] Ph. de Wailly det [p] // Pres. by Com. Ins. Ent. B. M. 1948–152 [p] // TYPE [h, red label]”, aedeagus is glued on label separately pinned: “CHINA, Foochow, KIENG, vi. 1936, M. S. Yang [h] // Brit. Mus. 1948–152 [h] // next to Polyphylla nov. sp. [h] Ph. de Wailly det [p]”, in BMNH.

#### Additional material examined.

CHINE Guangxi / Da Yao Shan / V. VI. 2008 / SINIAEV leg., 2 males and 1 female in DKCP.

#### Diagnosis.

**Figures 11–15.**
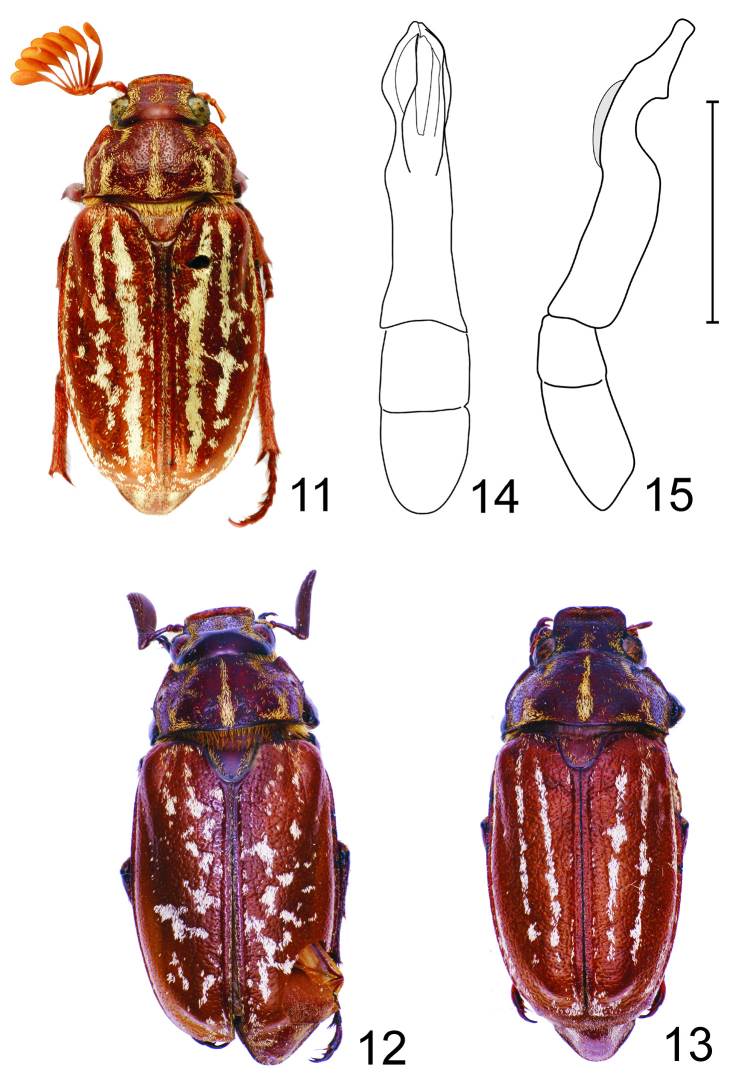
*Polyphylla (Granida) jessopi* **11** Habitus of holotype male (length 19.0 mm), dorsal view **12** Habitus of male (Guangxi, China, length 20.0 mm), dorsal view **13** Habitus of female (Guangxi, China, length 18.0 mm), dorsal view **14** Male genitalia, dorsal view **15** The same, right lateral view, shaded area indicates overlapping part of left paramere. Scale bar: 5 mm for Figs 14–15.

Scaly stripes on the elytra are partially fragmented (Figs 11, 13), rarely the elytra are completely maculate (Fig. 12). Antennomere 3 long and slender, three times longer than antennomere 2. Basal margin of pronotum convex medially. Male genitalia as in Figs 14–15.

#### Distribution.

Fukien and Guangxi provinces of China.

#### Remarks.

For a long time, *Polyphylla (Granida) jessopi* was known from single male only. Recently, Keith (2010) reported three additional specimens collected in Guangxi (China) with variable elytral pattern. Except of two specimens with the same pattern as the holotype, one male has maculate elytra. Such distinct variability in elytral pattern is very unusual in Palaearctic members of the genus *Polyphylla*.

### Polyphylla (Granida) phongsali

Zídek, 2006

http://species-id.net/wiki/Polyphylla_(Granida)_phongsali

Figs 16–19

Polyphylla phongsali  Zídek, 2006: 10, pl. 3.

#### Type locality.

“N. Laos, Phongsali, Gnoi-ou”.

#### Type material examined.

Holotype (male), labeled: “N. Laos, Phongsali, Gnoi-ou, Li Jingke VI-2003 [h] // BMNH (E) 2006–162 [h] // J. ZIDEK det. 2006 [p] Polyphylla phongsali Zídek [h] HOLOTYPE [p, red label]”, in BMNH.

#### Additional material examined.

LAO-NE, Hua Phan prov., ~20˚12′N, 104˚01′E, PHU PHAN Mt. 1500–1900m, 17.v.-31.vi. 2007, M. Brancucci leg., 1 male in ABCC; LAOS-NE, Houa Phan prov., 20˚13′09-19″N, 103˚59′54″-104˚00′03″E, 1480–1510m, PHOU PHANE Mt., 22.iv.-14.v.2008, Vít Kubáň leg., 1 male and 1 female in NMPC; LAOS-NE, Houa Phan prov., 20˚13′N 103˚59′E, Ban SALUEI village, 16.vi.2009, 1350 m, at light, Vít. Kubáň leg., 1 male in NMPC; LAOS-NE, Houa Phan prov., 20˚12-13.5′N 103˚59.5′-104˚01′E, Ban Saluei → Phou Pane Mt., 1340–1870 m, 1.v.-16.vi. 2009, Lao collectors leg., 1 male in NMPC; Laos, Houaphan prov., 38 km S of Sam Neua, Saluei 9.-22.5.2009, Martinů lgt. 1350–1900 m, 1 male in JZCP and 1 male in RSCV; Laos, Houaphan prov., 38 km S of Sam Neua, Saluei 9.-22.5.2009, Bednařík lgt. 1350–1900 m, 1 male in RSCV.

#### Diagnosis of female

**Figures 16–19.**
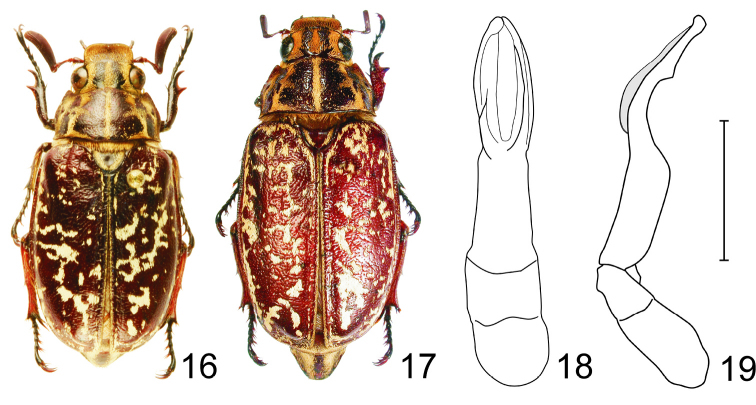
*Polyphylla (Granida) phongsali* **16** Habitus of male (Hua Phan, Laos, length 22.5 mm), dorsal view **17** Habitus of female (Hua Phan, Laos, length 28.0 mm), dorsal view **18** Male genitalia, dorsal view **19** The same, right lateral view, shaded area indicates overlapping part of left paramere. Scale bar: 5 mm for Figs 18–19.

(Fig. 17). Similar to male (Fig. 16), with the following exceptions. The length of the only known female specimen is 28.0 mm (except of pygidium), while the length of males varies between 21.5–24.5 mm. Anterior margin of clypeus only very feebly upturned, nearly flat. Antennal club pentamerous. Outer margin of anterior tibia distinctly tridentate. Upper apical spur of metatibia broad, flattened, blunt apically. Tarsal claws of all pairs of legs equal in length.

#### Collecting methods.

The female specimen was collected at light.

#### Diagnosis.

An easily recognizable *Polyphylla (Granida)* species because of its maculate elytra. Antennomere 3 long and slender, more than three times longer than antennomere 2. Basal margin of pronotum is almost straight against the scutellum, while convex in other *Granida* species. It is most likely to be confused only with some *Polyphylla (Granida) jessopi* specimens bearing the same maculate elytral pattern. *Polyphylla (Granida) phongsali* in average larger than *Polyphylla (Granida) jessopi* (the length of males varies between 21.5–24.5 mm versus 18.5–20.0 mm in *Polyphylla (Granida) jessopi*). Male genitalia as in Figs 18–19.

#### Distribution.

Northern Laos.

#### Remarks.

**Figure 20.**
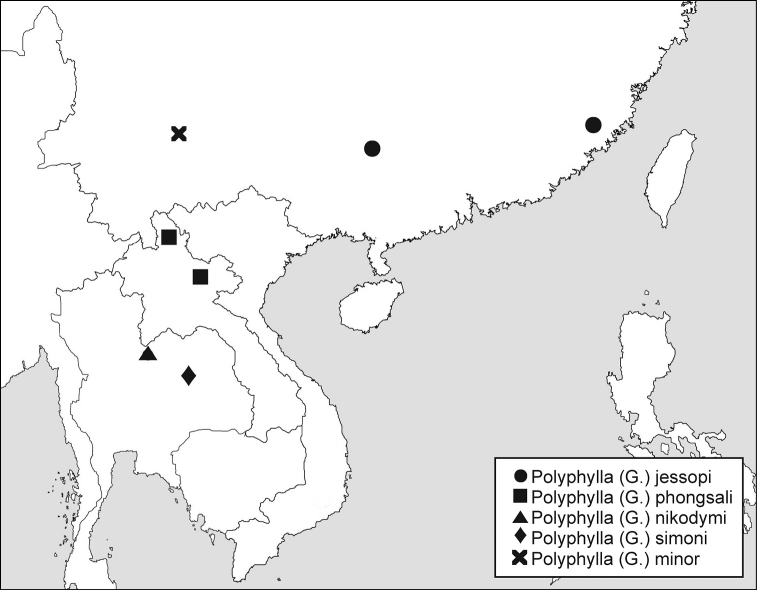
Distribution of *Polyphylla (Granida)* species in continental Asia. Because of imprecise locality data, distributional mark of *Polyphylla (Granida) nikodymi* in Myanmar is omitted.

The species was originally described from a single male. Here we recorded eight additional specimens from northern Laos (Fig. 20). The specimens with altitude data were collected between 1350–1900 m a.s.l.

## Supplementary Material

XML Treatment for Polyphylla (Granida) simoni

XML Treatment for Polyphylla (Granida) minor

XML Treatment for Polyphylla (Granida) nikodymi

XML Treatment for Polyphylla (Granida) jessopi

XML Treatment for Polyphylla (Granida) phongsali
